# Reach-To-Grasp Movements: A Multimodal Techniques Study

**DOI:** 10.3389/fpsyg.2018.00990

**Published:** 2018-06-15

**Authors:** Sonia Betti, Giovanni Zani, Silvia Guerra, Umberto Castiello, Luisa Sartori

**Affiliations:** ^1^Dipartimento di Psicologia Generale, Università di Padova, Padua, Italy; ^2^Centro Linceo Interdisciplinare Beniamino Segre, Accademia Nazionale dei Lincei, Rome, Italy; ^3^Padova Neuroscience Center, Università di Padova, Padua, Italy

**Keywords:** reach-to-grasp, transcranial magnetic stimulation, kinematics, MEP, EMG

## Abstract

The aim of the present study was to investigate the correlation between corticospinal activity, kinematics, and electromyography (EMG) associated with the execution of precision and whole-hand grasps (WHGs). To this end, motor-evoked potentials (MEPs) induced by transcranial magnetic stimulation (TMS), EMG, and 3-D motion capture data have been simultaneously recorded during the planning and the execution of prehensile actions toward either a small or a large object. Differences in the considered measures were expected to distinguish between the two types of grasping actions both in terms of action preparation and execution. The results indicate that the index finger (FDI) and the little finger (ADM) muscles showed different activation patterns during grasping execution, but only the FDI appeared to distinguish between the two types of actions during motor preparation. Kinematics analysis showed that precision grips differed from WHGs in terms of displayed fingers distance when shaping before object’s contact, and in terms of timing and velocity patterns. Moreover, significant correlations suggest a relationship between the muscular activation and the temporal aspects concerned with the index finger’s extension during whole-hand actions. Overall, the present data seem to suggest a crucial role played by index finger as an early “marker” of differential motor preparation for different types of grasps and as a “navigator” in guiding whole-hand prehensile actions. Aside from the novelty of the methodological approach characterizing the present study, the data provide new insights regarding the level of crosstalk among different levels concerned with the neuro-behavioral organization of reach-to-grasp movements.

## Introduction

A large amount of behavioral and neurophysiological studies have identified specific kinematic patterns and neural activations for grasping of differently shaped objects (for review, see Castiello, 2005). Effective grasping implicates the ability to coordinate multiple configurations of finger movements, depending on the properties of the object to be grasped (e.g., size, shape, and weight). This process first involves a progressive opening of the grip with straightening of the fingers during reaching, followed by a closure of the grip until it matches object size and shape. Precision grip (PG; i.e., the opposition of the thumb to the index finger) on small objects requires smaller hand aperture. Increasing object size, instead, lowers the spatial accuracy demands, permitting a larger grip to emerge in a whole-hand grasp (WHG; i.e., the opposition of the fingers to the palm). Interestingly, subpopulations of neurons in the primary motor cortex (M1) of non-human primates are active while conducting a PG, but not during a WHG (Muir and Lemon, 1983). This indicates that the control of fingertip actions with a PG engages neural circuits that are different to those engaged during the phylogenetically older WHG (Napier, 1980).

Despite the interest on motor preparation and execution of different types of grasps, the functional connection between these two processes still needs to be clarified (Prabhu et al., 2007). A very useful measure of motor planning is provided by the amplitude of the motor-evoked potential (MEP) in response to a standard single pulse of transcranial magnetic stimulation (spTMS) over M1 (Priori et al., 1998). Transcranial magnetic stimulation (TMS) was first introduced as a method to investigate the integrity of the corticospinal (CS) outflow from cerebral motor cortex to the spinal cord (Rothwell, 1997). The TMS pulses penetrate the skull and carry an electric stimulating current into the cortex. Depolarization of neurons is produced by virtue of an induced current, as prescribed by Faraday’s law (Epstein, 2008). In the motor area, action potentials leads to activation of pyramidal neurons, conduction of impulses to the spinal cord, and eventually to contraction of muscles on the contralateral side of the body (Davey, 2008). The M1 and its descending projection to the spinal cord in the CS tract, in particular, are crucial for the control of hand and finger movements (Muakkassa and Strick, 1979; Godschalk et al., 1984; Matelli et al., 1986; Dancause et al., 2006). Much of the work involving magnetic stimulation of the human motor cortex, therefore, has focused on electromyographic (EMG) responses in hand muscles during action execution (Lemon et al., 1995). Interestingly, the TMS pulses tend not to activate the pyramidal output neurons directly, but instead to stimulate the axons of neurons that synapse onto them. Thus, the size of the response produced by a given stimulus is sensitive to the excitability of synaptic connections within the cortex, giving an indirect measure of the excitability of intrinsic cortical circuits within the conscious brain (Quartarone et al., 2006).

In terms of action execution, motion-capture technology has allowed researchers to build up a detailed and complex picture of how action kinematics vary depending on the relationship between types of prehensile actions and intrinsic object properties (Gentilucci et al., 1991; Castiello et al., 1993, Castiello et al., 1996). In particular, the dimension of an object influences how it is manipulated, with the maximum grip aperture (MGA; i.e., the opening of the fingers while approaching the object) varying linearly as a function of the size of the object (Jeannerod, 1981; Marteniuk et al., 1990). Moreover, different types of grasping (i.e., PG and WHG) are characterized by different temporal patterns, with the time of MGA occurring earlier for PG than for WHG (Gentilucci et al., 1991).

Given the vast interest on the mechanisms underlying the execution of prehensile actions, here we specifically devised a multi-methodological study in order to unveil for the first time the relationship between the neural underpinning of motor preparation and the unfolding of hand shaping – as identified, respectively, by CS excitability, EMG, and kinematics. We recorded MEPs and EMG from two intrinsic hand muscles: the first dorsal interosseous (FDI) and abductor digiti minimi (ADM) during the planning and execution of precision and WHGs. Since FDI is a prime mover in PG, whereas the ADM abducts the little finger to open the hand in the WHG (Cattaneo et al., 2005; Davare et al., 2009, 2010; Cavallo et al., 2011), we predict facilitation effects for those muscles during the preparation and execution of the respective action sequences. Moreover, MEP literature also highlights FDI modulation during the observation of a WHG, in correspondence to the maximal finger aperture phase (e.g., Gangitano et al., 2001). This aspect might be crucial when considering potential correlations between FDI activity during action execution and kinematics. In addition to MEPS and EMG recording, motion capture was applied to measure hand kinematics. In terms of crosstalk between CSE, EMG, and kinematics, no firm predictions can be made given that this is the first study investigating the activity sequence from action preparation to action execution, at both the neural and behavioral levels.

## Materials and Methods

This experiment investigated the reciprocal contribution of CS activity, kinematics, and EMG associated with the preparation and execution of precision and WHGs.

### Participants

Twenty-five naïve volunteers (15 female and 10 male, aged between 21 and 30 years, mean age 23.92 ± 2.4 years) took part in the experiment. All participants were right-handed, as assessed with the Edinburgh Handedness Inventory (Oldfield, 1971), with normal or corrected-to-normal visual acuity. They were all screened for TMS exclusion criteria and for neurological, psychiatric, and medical problems (Wassermann, 1998; Rossi et al., 2009). The experiment was approved by the ethics committee of the University of Padua, in accordance with the Declaration of Helsinki (sixth revision, 2008). All participants gave their written informed consent and were financially compensated for their participation.

### Experimental Stimuli

**FIGURE 1:**
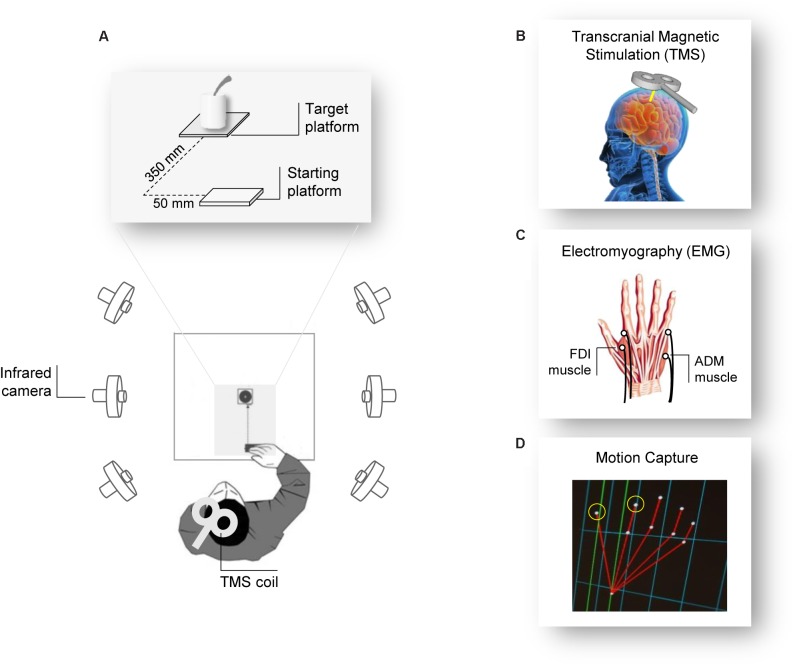
Experiment setup. **(A)** The participants were seated in front of a table surrounded by six infrared cameras. The participant’s hand was positioned in front of the object they had to grasp after the Go signal (TMS pulse). **(B)** The TMS coil was placed over the participant’s left M1 and **(C)** MEPs and EMG activity were measured from electrodes placed over the FDI and the ADM muscles of the right hand. **(D)** Two infrared reflective markers taped to the participant’s index and thumb fingers were used to track the right hand’s kinematics.

The participants sat comfortably in front of a table (∼90 cm wide, ∼90 cm long) upon which a cup (∼12 cm height, ∼9 cm diameter) with a spoon (∼20 cm long) inside it and a starting platform (∼2 cm wide, ∼4 cm long) were placed. The cup was positioned alongside the participants’ midsagittal plane at a 30 cm distance from the starting platform. Participants placed their right hand in pinch position on the starting button at the outset of each trial (**Figure 1A**).

### Procedure

Participants were tested individually in a single experimental session lasting 1 h. They were seated in a comfortable chair with the right elbow positioned on an adjustable armrest, the head on a fixed headrest, and the right hand’s ulnar styloid process laying on the starting platform with the hand in pinch position (**Figure 1A**). They were instructed to remain as still and relaxed as possible between each trial and to keep their eyes open. At the beginning of each block, participants were verbally instructed on the type of action they would have to perform. The order of the actions was counterbalanced between participants. TMS pulse served as a “Go” signal, after which participants were required to either (a) release the start button, reach and grasp the cup with a WHG, and lift it, or (b) release the start button, reach and grasp the sugar spoon with a PG, and move it. At the end of each action (WHG, PG), participants were requested to place the object at its original position and then return to the starting position. Participants performed a total of 32 trials, divided in four blocks of eight repetitions each; therefore, they performed 16 repetitions for each type of action. For each trial, right hand TMS-induced MEPs from the participants’ FDI and ADM muscles were recorded during action preparation, together with EMG activity and kinematics recordings during action execution.

#### Transcranial Magnetic Stimulation

Single-pulse TMS was administered using a 70 mm figure-of-eight coil connected to a Magstim Bistim2 stimulator (Magstim Co., Whitland, United Kingdom). Pulses were delivered on the participant’s left primary motor cortex (M1), in correspondence with the right hand representation. The coil was placed on the head with a 45° angle relative to the inter-hemispheric fissure, with the handle pointing laterally and caudally (Brasil-Neto et al., 1992; Mills et al., 1992; **Figures 1A,B**). The optimal scalp position (OSP), which is defined as the scalp position at which the minimum level of stimulation elicits the largest MEPs from both the ADM and the FDI muscles, was determined by moving the coil in approximately 0.5 cm steps around the presumed hand motor area. The OSP was then marked on a tight-fitting cap worn by the participants ensuring a correct coil placement throughout the experiment. During the experiment, the coil was held by a tripod and continuously checked by the experimenters to maintain a constant positioning with respect to the marked OSP. The stimulation intensity was then set at 120% of the rMT (see below). TMS stimulation was managed by E-Prime V2.0 software (Psychology Software Tools Inc., Pittsburgh, PA, United States).

#### Electromyographic Recording

Electromyographic activity was recorded through two pairs of surface Ag/AgCl electrodes (1 cm diameter) placed in a belly-tendon montage (**Figure 1C**). After skin cleaning, electrodes containing a small amount of water-soluble EEG conductive paste were placed and fixed on the target positions. The active electrode was placed over the muscle belly (determined by palpation during maximum voluntary contraction) and the reference over the proximal interphalangeal juncture. The ground electrode was positioned over the participant’s right wrist. The electrodes and wires were secured and positioned so that they did not restrict the participants’ movements. Skin impedance, evaluated at rest prior to beginning the experimental session, was considered of good quality when below the threshold level (5 Ω). Electrodes were connected to an isolable portable ExG input box linked to the main EMG amplifier for signal transmission via a twin fiber optic cable (Professional BrainAmp ExG MR, Munich, Germany). A high-pass filter of 30 Hz and a low-pass filter of 1000 Hz were applied to the raw myographic signal, which was amplified prior to being digitalized (5 KHz sampling rate), and stored on a computer for offline analysis. MEPs and EMG activity were recorded simultaneously from the FDI and ADM muscles of the participant’s right hand. We also determined the individual resting motor threshold (rMT) as the minimum TMS intensity able to produce MEPs with an at least ≥50 μV peak-to-peak amplitude in a relaxed muscle in 5 out of 10 consecutive pulses (Rossini et al., 1994) in the higher threshold muscle (ADM). rMT ranged from 28 to 52% (mean = 40.1%, SD = 5.5) of the maximum stimulator output. EMG recordings were managed by E-Prime V2.0 (Psychology Software Tools Inc., Pittsburgh, PA, United States) and Brain Vision Recorder (Brain Products BmbH, Munich, Germany) software.

#### Kinematics Recording

Movements were tracked using the 3-D optoelectronic SMART system (Bioengineering Technology and Systems, B| T| S|, Milan, Italy) equipped with six infrared cameras (sampling rate 60 Hz), placed in a semicircle at a distance of 1–1.2 m from the table (**Figure 1A**). Two semi-spherical reflecting markers (∼0.25 mm diameter) were attached to the participants’ right hand on the radial side of the index nail and on the ulnar side of the thumb nail (**Figure 1D**). The index finger and thumb markers served to measure the manipulation component of the grasping action. Cameras position, roll angle, focus, zoom, brightness, and threshold were set before the experimental sessions to optimize markers’ tracking. Static and dynamic calibrations were then performed for 3-D space reconstruction. For the static calibration, a three-axis frame of markers at known distance was placed at the center of the table, allowing to determine the spatial coordinate system. For the dynamic calibration, a three-marker wand was moved up and down several times parallel to each axis throughout the workspace of interest. The SD of the reconstruction error was below 0.3 mm for all the axes (*x*, *y*, and *z*).

### Data Analysis

#### MEP Data

Individual peak-to-peak MEP amplitudes (mV) were analyzed off-line using Brain Vision Analyzer (Brain Products BmbH, Munich, Germany). The MEP peak-to-peak amplitude for FDI and ADM muscles was determined as a measure of participants’ CS excitability. Trials in which any EMG activity greater than 100 μV was present in the 100 ms window preceding the TMS pulse were discarded to prevent contamination of MEP measurements by background EMG activity (<1%).

#### EMG Data

Electromyographic activity was analyzed offline using Brain Vision Analyzer (Brain Products BmbH, Munich, Germany). The EMG signal from the FDI and ADM muscles during action execution was rectified (Rectify function of the Brain Vision Analyzer software; no smoothing) and the area under the curve of the rectified EMG track (mV^∗^s) was calculated for each muscle and each trial to quantify muscular activity when executing the grasping actions (PG, WHG). To explore the variations of EMG activity over time for each type of action, EMG activity was measured within a time window starting 500 ms after the TMS-go signal pulse up to 4500 ms. This window was subdivided in four time bins of 1 s each. The four time bins were defined as follows: (*T*_1_) 500–1500 ms; (*T*_2_) 1500–2500 ms; (*T*_3_) 2500–3500 ms; and (*T*_4_) 3500–4500 ms. In order to better take into account possible time differences across participants, for each participant and type of action, we calculated a 1-s time window based on the time at which the maximum distance between the thumb and index finger was reached (TMGA, see the next paragraph), comprising 500 ms before and after it (*T*_individualized_).

#### Kinematics Data

Following kinematic data collection, the 3-D markers positions as a function of time were reconstructed, filtered (Butterworth filter with a 6 Hz cutoff), and analyzed by means of the SMART-D Tracker and SMART-D Analyzer software packages (B| T| S). Jeannerod (1981, 1984) coded grasping in terms of changes in grip aperture – the separation between the thumb and the index finger – and described two major components for prehensile behavior: the transport and the grasp components. The transport component brings the hand in the vicinity of the object and it is analyzed on the basis of the 3-D position of the wrist in time. The grasp component, instead, is concerned with finger pre-shaping during transport and finger closing around the object. Given that we aimed at investigating the crosstalk between finger muscles’ EMG and kinematics, analyses were confined to the grasp component. Notably, the FDI is an intrinsic hand muscle that receives the strongest cortical input as it closes around the object (Lemon et al., 1995) and it is specifically implicated in grip aperture (Jeannerod, 1981). The following kinematic parameters were then extracted for each individual movement to measure the manipulation component:

*Reaction times (RTs)*: The time at which participants released the start button after the “Go” signal.

*Maximum grip aperture*: The maximum distance reached by the 3-D coordinates of the thumb and index finger.

*Time of maximum grip aperture (TMGA)*: The time at which the distance between the 3-D coordinates of the thumb and index finger was maximum from movement onset.

*% Time of maximum grip aperture (TMGA%)*: The percentage of time at which the distance between the 3-D coordinates of the thumb and index finger was maximum with respect to grasping time.

*Maximum grip velocity (MGV)*: The maximum velocity reached by the 3-D coordinates of the thumb and index finger during grip aperture.

*Time of maximum grip velocity (TMGV)*: The time at which the tangential velocity of the 3-D coordinates of the thumb and index finger was maximum from movement onset.

*% Time of maximum grip velocity (TMGV%)*: The percentage of time at which the tangential velocity of the 3-D coordinates of the thumb and index finger was maximum with respect to grasping time.

#### Statistical Analysis

SPSS 23 (SPSS Inc., Chicago, IL, United States) was used for statistical analysis. A repeated-measure ANOVA (rmANOVA) with condition (PG, WHG) and muscle (FDI, ADM) as within-subject factors was performed on MEP amplitudes. An rmANOVA on EMG activity was performed with condition (PG, WHG) muscle (FDI, ADM) and time (*T*_1_–*T*_4_) as within-subject factors. Moreover, to deeply investigate inter-individual time variations in muscular activation, an rmANOVA on EMG activity was performed on *T*_individualized_ with condition (PG, WHG) and muscle (FDI, ADM) as within-subject factors. For kinematics parameters, the mean values for each parameter of interest (RT, MGA, TMGA, TMGA%, MGV, TMGV, and TMGV%) were determined for each participant and entered into separate rmANOVAs with action (PG, WHG) as within-subjects factor. The partial eta square (${\text{η}}_{\text{p}}^{2}$) value was calculated as an estimate of effect size. In the presence of significant interactions, *post hoc* comparisons were performed. To explore the crosstalk between MEP and EMG measures and between EMG and kinematics, correlations were computed using the Pearson correlation coefficient. Each *p*-value obtained was corrected with Bonferroni correction. A significance threshold of *p* < 0.05 was set for all statistical analyses.

## Results

### Grasp Preparation: MEP

**FIGURE 2:**
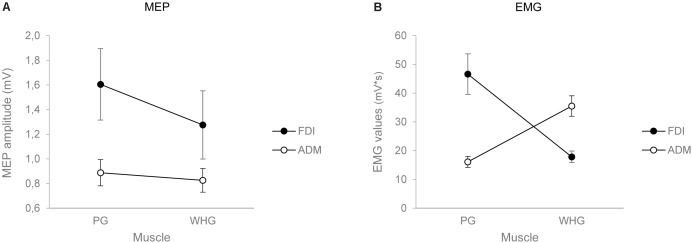
Graphical representation of the mean values for the MEP amplitude **(A)** and the EMG activity in *T*_individualized_ **(B)** for the FDI (black circles) and the ADM (white circles) muscles when participants performed either a PG or a WHG. Bars represent SE of the mean.

The ANOVA on MEP amplitudes showed a main effect of muscle [*F*_(1,24)_ = 6.037, *p* = 0.022, ${\text{η}}_{\text{p}}^{2}$ = 0.201], action [*F*_(1,24)_ = 8.847, *p* = 0.007, ${\text{η}}_{\text{p}}^{2}$ = 0.269], and a significant interaction of muscle by action [*F*_(1,24)_ = 8.556, *p* = 0.007, ${\text{η}}_{\text{p}}^{2}$ = 0.263]. *Post hoc* contrasts revealed that MEP amplitudes for the FDI muscle were higher while preparing a PG compared to a WHG (*p* = 0.004). Moreover, the preparation for a PG was characterized by an increase in MEP amplitudes of the FDI compared to the ADM muscle (*p* = 0.006). Results are graphically summarized in **Figure 2A**.

### Grasp Execution: Electromyography

**FIGURE 3:**
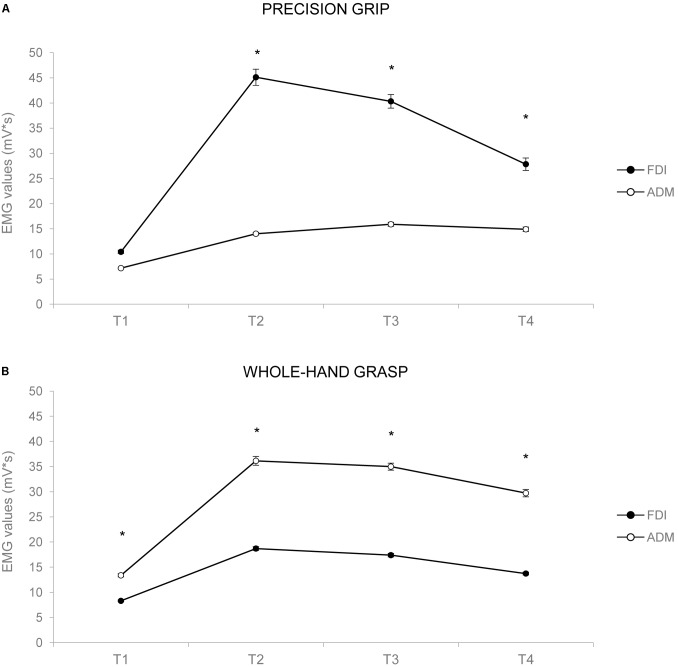
Graphical representation of the mean values for the EMG activity across the 1-s time bins (*T*_1_–*T*_4_) for the FDI (black circles) and the ADM (white circles) muscles during PG **(A)** and WHG **(B)**. Bars represent SE of the mean. Asterisks indicate statistically significant comparisons (*p* < 0.05).

The ANOVA on EMG activity across time bins (*T*_1_–*T*_4_) showed a main effect of time [*F*_(1,24)_ = 26.834, *p* < 0.001, ${\text{η}}_{\text{p}}^{2}$ = 0.528], a significant interaction of muscle by action [*F*_(1,24)_ = 40.843, *p* < 0.001, ${\text{η}}_{\text{p}}^{2}$ = 0.630], muscle by time [*F*_(1,24)_ = 2.849, *p* = 0.043, ${\text{η}}_{\text{p}}^{2}$ = 0.106], and muscle by action by time [*F*_(1,24)_ = 13.431, *p* < 0.001, ${\text{η}}_{\text{p}}^{2}$ = 0.359]. Results are graphically summarized in **Figure 3**. In PG trials, the FDI was more active compared to the ADM muscle at *T*_2_ (*p* = 0.001), *T*_3_ (*p* = 0.001), and *T*_4_ (*p* = 0.020; **Figure 3A**). In WHG trials, the ADM was more active compared to the FDI muscle throughout all the four time bins (*T*_1_–*T*_4_, *p*_s_ < 0.001; **Figure 3B**). FDI was more activated in PG than WHG from *T*_2_ to *T*_4_ (*p*_s_ < 0.020), whereas ADM was more activated in WHG than PG in all time bins (*T*_1_–*T*_4_, *p*_s_ < 0.001). Moreover, the FDI muscle during both PG and WHG was less activated during the first time bin in *T*_1_ compared to later time bins (i.e., *T*_2_–*T*_4_, *p*_s_ < 0.05), and during *T*_4_ compared to *T*_3_ (*p* = 0.006 and *p* = 0.021, respectively). Similarly, the ADM muscle during both PG and WHG was less activated during the first time bin in *T*_1_ compared to *T*_2_–*T*_4_ (*p*_s_ < 0.013). The ANOVA on EMG activity at *T*_individualized_ showed a significant interaction of muscle by action [*F*_(1,24)_ = 39.706, *p* < 0.001, ${\text{η}}_{\text{p}}^{2}$ = 0.623]. In PG trials, the FDI was more active compared to the ADM muscle (*p* < 0.001); conversely, in WHG trials, the ADM was more active than the FDI muscle (*p* < 0.001). FDI muscle showed a greater activation in PG compared to WHG trials (*p* < 0.001), whereas the ADM muscle was more activated in WHG than in PG trials (*p* < 0.001). Results are graphically summarized in **Figure 2B**. As concerns the temporal distribution of *T*_individualized_, we calculated that for PG trials, the TMGA occurred within the first time bin (*T*_1_) for the 20% of participants and within the second time bin (*T*_2_) for the 80% of participants. For WHG trials, the TMGA occurred within the first time bin (*T*_1_) for the 12% of participants, within the second time bin (*T*_2_) for the 76% of participants, and within the third time bin (*T*_3_) for the 12% of participants. Overall, the TMGA occurred within *T*_2_ for the most of the participants.

### Grasp Execution: Kinematics

**FIGURE 4:**
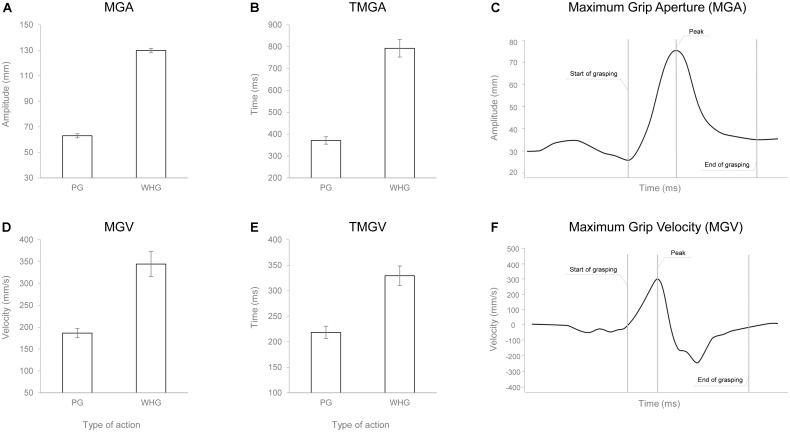
Graphical representation of the mean values for MGA **(A)**, TMGA **(B)**, MGV **(D)**, and TMGV **(E)** when participants performed either a PG or a WHG. Bars represent SE of the mean. Example of the measure of the MGA **(C)** and MGV **(F)** peaks from a representative participant.

The ANOVA on RT did not show any statistically significant effect [*F*_(1,24)_ = 0.010, *p* = 0.921, ${\text{η}}_{\text{p}}^{2}$ < 0.001]. The ANOVA on MGA showed a main effect of action [*F*_(1,24)_ = 1356.217, *p* < 0.001, ${\text{η}}_{\text{p}}^{2}$ = 0.983], with WHG requiring a greater hand aperture compared to PG due to different object sizes (**Figures 4A,C**). The ANOVA on TMGA showed a significant main effect of action in both absolute [*F*_(1,24)_ = 156.387, *p* < 0.001, ${\text{η}}_{\text{p}}^{2}$ = 0.867; **Figure 4B**] and relative [*F*_(1,24)_ = 82.637, *p* < 0.001, ${\text{η}}_{\text{p}}^{2}$ = 0.775] terms, with a delayed peak of MGA for the WHG compared to the PG. The ANOVA on MGV showed a significant main effect of action [*F*_(1,24)_ = 45.742, *p* < 0.001, ${\text{η}}_{\text{p}}^{2}$ = 0.656], with a faster grip aperture for the WHG compared to the PG (**Figures 4D,F**). The ANOVA on TMGV showed a significant main effect of action in both absolute [*F*_(1,24)_ = 38.093, *p* < 0.001, ${\text{η}}_{\text{p}}^{2}$ = 0.61; **Figure 4E**] and relative [*F*_(1,24)_ = 61.356, *p* < 0.001, ${\text{η}}_{\text{p}}^{2}$ = 0.72] terms, with an earlier velocity peak for PG execution compared to the WHG.

### Correlations Between MEP and EMG

No significant correlations emerged when correlating MEP amplitudes with the EMG activation neither during *T*_individualized_ (*p*_s_ > 0.05) nor for *T*_1_–*T*_4_ bins (*p*_s_ > 0.05).

### Correlations Between EMG and Kinematics

**FIGURE 5:**
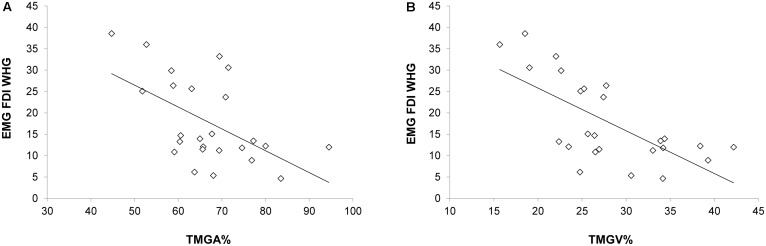
Negative correlations between the EMG activity (*T*_individualized_) in the FDI muscle during the WHG and **(A)** the time at which the grip aperture was maximum from movement onset and **(B)** the time at which the velocity of grip aperture was maximum from movement onset.

When correlating EMG activations during *T*_individualized_ and kinematics, negative correlations emerged between the EMG activity of the FDI muscle and both the TMGA% and TMGV% [*r*_(23)_ = -0.547, *p* = 0.019, **Figure 5A**; *r*_(23)_ = -0.676, *p* < 0.001, **Figure 5B**, respectively]. In particular, an increased activation of FDI occurred when MGA (TMGA%) and peak velocity of grip aperture (TMGV%) were anticipated. Notably, when performing the same correlations for all the time bins (*T*_1_–*T*_4_), we found that only for T_2_ negative correlations emerged between the EMG activity of the FDI muscle and both the TMGA% and TMGV% [*r*_(23)_ = -0.554, *p* = 0.033; *r*_(23)_ = -0.593, *p* = 0.014, respectively]. No other significant correlations emerged for either the FDI muscle during PG or the ADM muscle during PG or WHG (*p*_s_ > 0.05).

## Discussion

The aim of the present study was to unveil the relationship between the neural underpinning of motor preparation and the unfolding of hand shaping – as identified by CS excitability, EMG, and kinematics, respectively. Results confirmed previous literature on motor preparation showing that planning a PG entailed an increased MEP amplitude in the FDI muscle with respect to the ADM muscle (not recruited in the PG) and with respect to the preparation of a WHG, that requires minor intervention from the index finger muscle. Results from the EMG extended these results to the ADM muscle and showed that during PG trials the FDI muscle was more active compared to both the ADM and during WHG trials. Moreover, during WHG trials, the ADM muscle was more active compared to both the FDI and to muscle activity during PG trials. No significant correlations emerged when correlating MEP amplitudes during action preparation with EMG activity during action execution. In terms of kinematics, results confirmed the linear relationship between grip aperture and object size, with smaller aperture for PG and larger for WHG (Gentilucci et al., 1991). Moreover, an early peak of MGA and MGV was found for the PG compared to the WHG, indicating that performing a PG required a more precise determination of contact points, resulting in an anticipated hand aperture (Gentilucci et al., 1991). When correlating EMG activations and kinematics of grasping during action execution, significant correlations emerged for the FDI muscle during WHG. In particular, the more MGA and MGV were anticipated, the more FDI was activated.

### Different Planning Strategies for Different Types of Grasping

When the participants prepared for a PG, the muscle specifically involved in this action (the FDI) was facilitated to a greater extent than when preparing a WHG. In contrast, the ADM did not lead to significantly larger MEPs when preparing a WHG relative to preparing a PG. Interestingly, this a-specific pattern for the ADM muscle is convergent with previous findings during finger observation (Kaneko et al., 2007; Naish and Obhi, 2015). Hence, it is possible that the motor representation of the ADM is simply weaker/smaller than that of the FDI, being the former more frequently activated during, for instance, pointing movements or in synergy with the thumb to grasp and manipulate objects. In contrast, the little finger abduction is a relatively infrequent movement. A feature of the current design (and most studies of this type) that must be considered is that a single hot spot was chosen for stimulating both the FDI and ADM cortical maps and the intensity of stimulation was set based on the intensity required to elicit responses in the less excitable (higher threshold) muscle (ADM). So, it is possible that the FDI muscle might have been “over-stimulated,” with respect to its motor threshold, compared to the ADM. Both these hypothesis would be confirmed by the fact that MEPs elicited in FDI were greater than those triggered in ADM. Moreover, the WHG is phylogenetically older than the PG (Napier, 1980) and requires a different temporal unfolding, with a tardive abduction of the little finger on the object (Castiello et al., 1993). In contrast, the PG requires a precocious maximum aperture, which might be specified in advance in order to anticipate the closing phase. To sum up, it is possible that differences in motor representations, cortical maps, relative stimulation intensity, and temporal recruitment could have influenced our pattern of results, and this is a factor to bear in mind for future studies in both action preparation and execution.

### Dissociating Planning and Online Control

Planning and online control of action are two specialized processes serving different purposes and utilizing distinct visual representations (Glover, 2004). Choosing an appropriate motor plan depends on perceived information about the object and final goals. In particular, the planning process involves three aspects: (i) perceiving task-specific object properties; (ii) selecting a grasp strategy; and (iii) planning a hand location and orientation. Online control, instead, is feedback-based and it takes place during movement execution. Are these two processes independent one from each other? This is a highly controversial issue (Goodale and Westwood, 2004). Since no correlation was found here between MEPs and EMG, we might presume that a dissociation takes place between motor preparation and action execution. We must be cautious in interpreting these results, though, since no modulation was shown in the MEPs recorded from the ADM muscle. In this connection, we propose a more cautious approach when choosing the target muscles in future studies.

### The Index Finger Pattern

As concerns the significant correlation between the myographic and the kinematic components of the index finger movement during the WHG, we might advance a specific hypothesis. In a previous study with a similar whole-hand grasping task (Sartori et al., 2011), we demonstrated that participants adopted a particular motor pattern depending on the end-goal: the index finger tended to move away from the surface of the stimulus during the more demanding condition (i.e., pouring compared to moving). This strategy possibly allows for greater control (stabilizing mechanism) when stimulus dynamics become increasingly difficult (see also Crajé et al., 2011). To some extent, the index finger can be regarded as a “navigator” during computation of a hand trajectory toward a target (Sartori et al., 2011; Ansuini et al., 2015). Careful placement of the digits driven by the index finger is considered a prerequisite for a stable grasp (Kinoshita et al., 1995; Santello and Soechting, 2000). The present results, showing a correlation between index finger muscular activity and kinematics, specifically occurring within the time window including the MGA, seem to indicate that maximum aperture is the crucial event leading to index finger modulation. Notably, here grip aperture is much more expanded during the WHG with respect to the PG (12.99 vs. 6.3 cm, respectively). The greater finger extension needed to perform the WHG, therefore, might signify that the index finger act as a “navigator” and this might be the key to understand why we found a correlation only for whole-hand actions. To conclude, these results seem to suggest that the index finger may play a crucial role in driving the grasp component of whole-hand prehensile actions.

### Theoretical Implications

Over the past two decades, neuroscience research has largely modified the traditional view of the motor system. The simultaneous discovery of mirror neurons in the ventral premotor cortex of macaques (di Pellegrino et al., 1992) and the application of TMS to the human primary motor cortex (M1) during action observation (Fadiga et al., 1995) gave birth to the hypothesis of a neural system matching action observation and execution in humans as well as in monkeys. Nowadays, a considerable amount of data suggests that EMG responses in hand muscles recorded while an object is grasped exactly replicate the pattern of MEPs elicited by spTMS during action observation (e.g., Gangitano et al., 2001, 2004; Fadiga et al., 2005; De Stefani et al., 2013; Naish et al., 2014). So far, the combined TMS/MEP technique has taken research on the perception–action coupling mechanism a step further, producing original data with regard to the observation–execution matching system. Specifically, it has answered the questions of how and when observing another person’s actions produces motor facilitation in an onlooker’s corresponding muscles. In the light of this massive literature, the present data suggest a more cautious approach. In particular, the lack of correlation between MEPs and EMG and the poor sensitivity of ADM muscle must be taken into account in future studies.

## Conclusion

The present results confirm and extend the existing literature on motor preparation and execution indicating that the considered measures reliably distinguish between precision and whole-hand grasping actions. Moreover, significant correlations suggest a crosstalk between the muscular activation and the temporal unfolding of hand shaping. These findings offer new insights regarding the organization of reach-to-grasp movements and might have relevant translational implications on the literature concerning motor preparation and action execution.

## Author Contributions

SB and LS designed the study. SB, GZ, and SG collected, analyzed, and interpreted the data. SB, GZ, and LS wrote the manuscript. UC critically revised the manuscript.

## Conflict of Interest Statement

The authors declare that the research was conducted in the absence of any commercial or financial relationships that could be construed as a potential conflict of interest.
